# The illness management and recovery program: a contribution to recovery-oriented secondary mental health services

**DOI:** 10.1186/s12913-025-12907-2

**Published:** 2025-05-24

**Authors:** Kristin B. Ørjasæter, Tone Winnem, Kristin Heiervang, Ottar Ness, Kim T. Mueser

**Affiliations:** 1 Faculty of Nursing and Health Science, Division of Public Health and Community Participation, Nord University, Namsos, 7800 Norway; 2 WSO- We Shall Overcome. User and Interest Organization for Human Rights, Self-determination, and Dignity in Mental Health, Oslo, Norge; 3 Department of Research and Development, Division of Mental Health Services, Akershus University Hospital, Lørenskog, Norway; 4 Department of Education and Lifelong Learning, Norwegian University of Science and Technology, Trondheim, Norway; 5 Center for Psychiatric Rehabilitation, Departments of Occupational Therapy and Psychological & Brain Science, Boston University, Boston, MA USA

**Keywords:** Evidence-based practice, Focus group, Mental health practitioner, Mental illness, Psychosocial- treatment, Recovery

## Abstract

**Background:**

In recent decades, mental health services have been challenged to offer evidence-based practices (EBPs) that are person-centered, human rights- and recovery-oriented. The Illness Management and Recovery (IMR) program aims to promote recovery and enhance individuals’ ability to live well. Investigating the IMR program from the perspective of mental health practitioners is crucial to improve recovery-oriented care and optimize program delivery.

**Methods:**

This study aimed to develop knowledge whether IMR, as an EBP, can function as a recovery-oriented practice (ROP) within secondary mental health care services. Seven focus groups were conducted, each consisting of two to eight mental health practitioners with different professional backgrounds and experiences. Altogether, 36 practitioners from five mental health hospitals and district psychiatric centers in Norway participated. Reflexive thematic analysis was used to analyze the data.

**Results:**

The results show that IMR (a) actively facilitates the alignment of coping mechanisms with patients’ lives, and (b) emphasizes a recovery-oriented approach in the delivery of services. Mental health practitioners perceive IMR as inherently recovery-oriented. Although ROPs and EBPs are often considered to function in opposition, to the practitioners in our study they were neither contradictory nor mutually exclusive but complementary and with the potential to bring out the best in each other to support patients’ recovery processes.

**Conclusion:**

IMR, as an evidence-based, recovery-oriented program, bridges psychiatry and mental health recovery. However, challenges arise from its illness-oriented approach and language, as well as its emphasis on individual recovery aspects. While IMR underscores the social aspects of recovery, it is essential to highlight the social, and societal factors in individuals’ recovery processes within the program.

**Supplementary Information:**

The online version contains supplementary material available at 10.1186/s12913-025-12907-2.

## Introduction

To promote the recovery process of individuals’ with a mental illness and strengthen their ability to live well with mental illness in their communities, several psychosocial evidence-based practices (EBPs) for mental health services have been developed [1, 2], including assertive community teams (ACT), flexible assertive community treatment (FACT), supported employment (SE), and illness management and recovery (IMR). In this article, we explore practitioners’ perspectives on IMR as a recovery-oriented program in the context of secondary mental health services in Norway.

Recovery from mental illness has traditionally been understood as the elimination of symptoms and a return to normal functioning [3]. In the 1980s, however, Deegan [4] proposed a different conceptualization of recovery as the process of living a worthwhile and meaningful life with a mental illness, despite the presence of symptoms. She underscored that most people with a psychiatric illness have the same aspirations as those without one, such as to live, work and love in a community in which one makes a significant contribution [4]. Later, Anthony [5] introduced the term *personal recovery* to describe recovery as a deeply personal, unique process of changing one’s attitudes, values, feelings, goals, skills, and/or roles, which has become the most cited definition of *recovery* to date. However, with the rise of neoliberal influences, the emphasis in the mental health field has increased focus on individuals’ personal responsibility for their own health and well-being. This focus has often neglected recognition of the social determinants of mental health and the importance of contextual understandings in promoting recovery [6]. Several researchers have argued that recovery should be viewed not only as a personal process but also as a social one [6–10]. Topor et al. [6] argue for a need to incorporate the social, material, and contextual aspects of recovery more clearly and have suggested a new definition:

*“Recovery is a deeply social*,* unique*,* and shared process in which an individual’s living conditions*,* material surroundings*,* social relations*,* and sense of self evolve. In that light*,* recovery is about striving to live satisfying*,* hopeful*,* and reciprocal lives*,* even despite threats*,* stressful social situations*,* and distress”* [6].

In this study, we acknowledge the critique of viewing recovery solely as a personal process and thus consider it both personal and social.

### Recovery-oriented practices and evidence-based practices

Recovery-oriented practices (ROPs) refer to approaches where professionals collaborate with people with mental illness to support their recovery journeys [11], where the focus is on the individual’s needs and active involvement in their own treatment [12]. Davidson and colleagues [13] have defined ROPs as person-centered, strengths-based, collaborative, and empowering [13]. Similarly, Farkas et al. [14] believe that recovery can be promoted when the practices are characterized by program structures (i.e., mission, policies, procedures, record keeping and quality assurance) and staffing concerns (i.e., selection, training, and supervision) that align with the four key values of recovery: person-orientation, person involvement, self-determination, and personal growth. Le Boutillier, et al. [15] have further developed a conceptual framework outlining the key characteristics of ROPs: promoting citizenship, organizational commitment, supporting personally defined recovery and working relationships. Often, ROPs are described as best practices, based on expert opinion, rather than evidence [16].

On the other hand, evidence-based practices (EBPs) integrate best researched practices and clinical expertise with patient values. EBPs are standardized interventions that have been shown to be effective in rigorous research to improve outcomes that patients value [17]. The best available scientific evidence is preferably from randomized controlled trials evaluating their effectiveness comparing the intervention to alternative practices or to no intervention [18]. As EBPs require clinical expertise and judgement to implement and are attuned to the patients’ specific goals and preferences, they are not rigid, one-size-fits-all approaches to treatment. While standardized interventions can provide much of the structure and content of an intervention, and prescribe specific methods for teaching and providing supports, their delivery must be tailored to each patient’s unique circumstances, goals, and preferences by the mental health practitioner [19].

The relationship between ROPs and EBPs has been a topic of considerable debate [13]. Davidson et al. [13] proposed several possible relationships between the two. Some ROPs can be seen as evidence-based if they are sufficiently standardized for rigorous testing and found to be effective at improving important outcomes. However, others view ROPs and EBPs as incompatible due to their divergent focuses and methodologies [20–22]. Some Norwegian recovery researchers question the compatibility of standardized protocols and quantifiable measures, such as symptom reduction and clinical outcomes from randomized controlled trials, with ROPs. They also doubt the person-centeredness and individual tailoring in EPBs based on these practices [21]. These researchers argue that EBPs primarily rely on quantitative, experimental research adhering to a scientific ideal, which cannot adequately study ROPs, as measures related to subjective experiences and personal narratives, central to ROPs, are harder to quantify [21, 23]. Bøe [23] advocates for a wider interpretation of evidence to include more user and insider perspectives, aligning more with ROPs. Despite these concerns, it is possible for some EBPs to incorporate elements of ROPs particularly when they are adapted to include person-centered approaches and subjective measures, such as hope, control, self-agency, self-efficacy and a sense of defining and setting personal goals [13].

### The IMR program

IMR is an EBP aimed at empowering individuals with mental illness to develop and practice strategies to manage their conditions and both identify and work toward achieving personal recovery goals [24–27]. A starting point for psychosocial treatment programs is the belief that people with serious mental illness can play a decisive role in own recovery processes [26, 28]. For that reason, IMR practitioners work collaboratively with individuals to help them learn and put their illness management skills in action.

IMR involves using three different teaching approaches (educational, motivational and cognitive-behavioral) and incorporates five empirically supported illness management strategies (psychoeducation, relapse prevention, behavioral training, coping skills, and social skills) [25]. The theoretical underpinnings of IMR are: (1) the stress–vulnerably model, which posits that the course and outcome of severe mental illness is determined by the dynamic interaction between biological vulnerability, stress, and coping; and (2) the transtheoretical model, which maintains that the motivation to change develops across five stages—precontemplation, contemplation, preparation, action, and maintenance—and that facilitating change requires stage-specific interventions [25, 26]. IMR consists of 11 modules, listed in Table 1, and a workbook with educational handouts has been created to teach individuals with mental illness, either individually or in groups, weekly for 10 months or more intensively for 4–5 months. For IMR practitioners, including trained mental health practitioners and peer specialists, a toolkit has also been developed alongside fidelity checklists to guide the implementation of IMR [27, 29].

**Table 1:** Modules in the illness management and recovery program

Module	Title
Module 1	Recovery Strategies
Module 2	Practical Facts About Mental Illness
Module 3	Practical Facts About Substance Use Disorders
Module 4	The Stress–Vulnerability Model
Module 5	Coping With Stress
Module 6	Building Social Support
Module 7	Using Medications Effectively
Module 8	Coping With Problems and Symptoms
Module 9	Healthy Lifestyles
Module 10	Developing a Plan for Staying Well
Module 11	Getting Your Needs Met in the Health Care System

Most previous research on IMR has employed quantitative methods to assess its effectiveness [30–33] and implementation of IMR [34–37]. Qualitative studies have demonstrated that IMR aids patients and practitioners in setting individual goals [38, 39] and has highlighted that IMR’s core components – such as symptom management and peer sharing – enhance recovery processes [40]. Additionally, qualitative research indicates that the person-centered approach used by IMR practitioners’ fosters a good therapeutic alliance [41]. Despite these insights, information about practitioners’ experiences delivering IMR in hospital and district psychiatric centers remains scarce.

### Aim and research question

The aim of this study was to shed light on whether illness management and recovery (IMR), as an EBP, can function as an ROP within secondary mental health services in Norway from the practitioners’ perspective. Thus, we sought to develop an understanding of whether IMR can contribute to recovery-oriented outcomes and were guided by this. The research question: *In what ways does the delivery of Illness Management and Recovery impact the professional practices*,* attitudes*,* and beliefs of mental health practitioners in secondary mental health services?*

### Context of the study

Norway has a health care system with primary and secondary levels [42]. The primary level encompasses municipal services in mental health care, such as care homes, day centers, municipal mental health- and addiction teams and low-threshold mental health services. Their focus is on early intervention and community-based support [43]. The secondary level becomes relevant when patients require additional support beyond what primary healthcare can provide. At this level, health institutions offer a range of services, including outpatient clinics, day care units, flexible assertive community treatment and general psychiatric units. These services cater to patients with limited daily functioning, symptoms of psychosis, or who are a risk of harm to self or others [44]. Noteworthy features of secondary care services include timely treatment of mental illness, a focus on patient safety, and disease-oriented approach with an emphasis on risk management [11]. Additionally, when voluntary measures prove ineffective, coercive treatment is also available within secondary services [45]. The provision of specialized mental health services and interdisciplinary specialized substance use treatment (TSB) in hospitals and district psychiatric centers is overseen by four regional health authorities [43]. Although practitioners at both levels were offered IMR training, our study included only secondary-level practitioners.

Norway has the highest number of psychiatrists per capita globally (WHO) and ranks among the highest in numbers of psychologists and other mental health practitioners [46]. However, user-organizations, clinicians and politicians have questioned whether service delivery can be person-centered, human-rights-based, and recovery-oriented [47, 48]. Norwegian authorities are calling for radical cultural, attitudinal, and organizational leadership in mental health care [49, 50]. While the integration of research-based, clinical, and experiential knowledge is emphasized for effective treatment in secondary services, significant disagreement exists regarding their relative importance [47]. Criticism and doubt about treatments offered in Norway have proliferated to the point that even those claimed by mental health practitioners to be most effective, are not necessarily to be trusted (Reitan and Lien, 2024) or desired by patients [47]. This has led to a polarized debate [47, 51, 52], where critics have often been labeled as holding anti-psychiatric attitudes [46, 53], while the focus on developing efficient EBPs is criticized for conflicting with recovery-oriented principles [21].

## Methods

This qualitative study is based on a phenomenological approach [54], building on the assumption that the world visible to the individual is the real world [55]. Data were based on the “Illness Management and Recovery in Specialized Mental Health Care: Experiences of Professionals” project in Norway, which focusing on mental health practitioners’ understandings of IMR and their experiences of delivering it to people living with mental illness [39, 56].

### Recruitment and sampling

To gain an overview of secondary-level services that offer IMR, we initiated contact with the Norwegian IMR Network. This network serves as a national association for those who are interested in or involved in delivering IMR. Its primary objective is to promote the implementation and advancement of the IMR program in Norway. The network compiled a list of institutions operating at the secondary level. From this list, we selected specific Institutions based on several criteria: (a) geographical spread to ensure representation across Norway, (b) duration of IMR provision and (c) degree of expertise based on experiences of providing individual and group IMR treatment at the secondary level. Communication with the management at five secondary-level services was established by email or phone calls, and managers at all five services agreed to participate in the study. The managers also provided information to their staff and delegated the responsibility for recruitment and booking rooms if they could not do so themselves.

Practitioners in the study had to meet two criteria: (a) be employed as a mental health practitioner in outpatient or inpatient mental health care in a mental hospital or district psychiatric center, and (b) have experience delivering IMR (Table 2).

**Table 2:** Background information on participants (*N* = 36)

Sex	Female	27
	Male	9
Profession	Nurse	19
	Social worker	5
	Doctor or psychologist	4
	Occupational therapist or educator	4
	Other mental health professional	4
Workplace	Inpatient services	23
	Outpatient services	13
Years delivering IMR	< 2 years	10
	2–5 years	6
	> 5 years	20

Of the 36 practitioners included, 13 worked in outpatient services (i.e., psychosis outpatient clinic, aftercare outpatient clinic, FACT, and day care clinic), and the other 23 worked in inpatient services (i.e., general psychiatric unit, rehabilitation unit, and specialized psychiatric units for psychosis). Practitioners working in outpatient services held weekly IMR group sessions of closed-enrollment groups over a period of 9–10 months, whereas practitioners in inpatient services usually held open-enrollment group IMR sessions several times a week with rotating facilitators. Long-term inpatients tended to complete the entire IMR program in open-enrollment groups during their stay. Short-term inpatients (hospitalized for less than three months) received two to four modules (specifically, 2, 4, 5, and 7). Short-term inpatients who wanted to complete IMR were referred to an IMR program in outpatient services at the hospital or a district psychiatric center or in primary-level service with IMR.

### Data collection

Focus groups were formed to assess variations in practitioners’ understanding of IMR and experiences delivering it to people with mental illness. These focus groups are particularly well-suited for capturing shared meanings, interpretations, and interactions [57]. By gathering practitioners with firsthand experience, these focus groups facilitate in-depth dialogues and provide insights into their personal experiences, which are crucial for understanding the phenomenon being studied. Altogether, seven focus groups were conducted in the fall of 2021 by the first author. Each focus group consisted of two to eight practitioners in different professions. Because our primary aim was to promote discussion between practitioners, the researcher played the role of facilitator, not interviewer [58]. Hence, a discussion guide was created for the focus groups, including questions such as “Could you describe what IMR is and aim of the treatment program?”, “What do you perceive as the core of IMR?”, “How do you as staff experience working with IMR?” and “Could you share experience or thoughts on how IMR treatment aligns or conflicts with your understanding of treatment, roles, values, and flexibility?” During the focus group, the researcher posed open-ended questions and encouraged practitioners to freely share their experiences. The researcher aimed to avoid interruptions or steering the conversation, instead noting emerging themes and questions in a notebook for subsequent exploration.

The focus groups ranged from 81 to 98 min in length. All groups were audio-recorded and transcribed verbatim by a professional transcriber. Practitioners’ names were each assigned a research number (e.g., P1, P2, and P3) to protect their anonymity.

### Data analysis

Our data analysis was conducted through reflexive thematic analysis, adhering to the six-phase framework as proposed by Braun & Clark [59]. This interpretative approach to qualitative data analysis is instrumental in identifying and examining thematic patterns within a dataset [59] thereby foregrounding the researcher’s active role in the co-construction of knowledge [60]. While reflexive thematic analysis does not subscribe to any pre-existing theoretical framework, it necessities an explicit articulation of the chosen theoretical stance [59]. Embracing a phenomenological position entails a commitment to reporting the experiences, meanings, and realities of the participants as they perceive them. In phase 1, the transcribed interviews were read by the first author several times to become familiar with the data. In that process, an open, inductive approach to the data was attempted, and a list of ideas and thoughts about the data were jotted down. Phase 2 involved producing initial codes, while phase 3 focused on searching for themes. The initial codes were grouped and collated into potential main themes and subthemes and visualized in mind-maps using the software program Mindjet Manager [61], after which a meeting to discuss themes with the coauthors was arranged. Phase 4 involved reviewing and refining the themes. All collated extracts for each theme were read critically to determine whether they appeared to form a coherent pattern. Themes lacking coherence were refined, merged, or deleted. The themes were named: IMR (a) makes it about life, (b) is a structured program that “tunes in” to the individual, and (c) balances illness and social aspects. In phase 5, all themes (Table 3) were reassessed for coherence and overlap, and each theme was evaluated in relation to the others. This process resulted in new definitions and names for each main theme. The three main themes from phase four were reduced to two, with two sub-themes developed for each. Last, phase 6 involved producing this article, supported by quotations and an analysis addressing the research question.

**Table 3:** Overview of main themes and sub-themes

Main Theme	Sub-themes
IMR actively facilitates the alignment of coping mechanisms with patients’ lives	A program managing life
	Tailored to individual needs
IMR emphasizes a recovery-oriented approach in the delivery of services.	Balance between illness management and wellness promotion
	Integration of individual, social and health promoting perspectives

### Ethical considerations

The study was conducted in accordance with the Declaration of Helsinki and approved by the Norwegian Centre for Research Data (No. 2021/200019). Before the focus group interviews commenced, practitioners signed an informed consent and were given both written and oral information about the study and their rights as practitioners.

## Results

In terms of our research question, *“In what ways does the delivery of Illness Management and Recovery impact the professional practices*,* attitudes*,* and beliefs of mental health practitioners in secondary mental health services?”* our analysis revealed that IMR: (a) actively facilitates the alignment of coping mechanisms with patients’ lives and (b) emphasizes a recovery-oriented approach in the delivery of services.

### IMR actively facilitates the alignment of coping mechanisms with patients’ lives

This theme of IMR conveys that it actively shapes practitioners’ approaches, enhancing their understanding and assisting patients in living dignified and meaningful lives despite daily challenges. Practitioners agreed that IMR is a comprehensive learning program centered on life in general and everyday life in particular. As expressed by P3, “When someone asks me what [IMR] is, I say, “It’s a learning program about and for life, or *psychoeducation* as it’s called in professional language”. The program adapts to each patients’ unique experiences and perspectives´, guiding practitioners to delve into patients’ understandings of their ailments, past management efforts, life priorities and aspirations for change with professional support. Practitioners focus on equipping patients with coping strategies and fostering a supportive environment that promotes experimentation and learning, aiming to achieve a harmonious balance between managing their conditions and maintaining everyday life. As P14 succinctly put it: It’s a program where you learn how to deal with everyday life while living with a serious mental illness and associated symptoms. The goal [of the program] is not to become symptom-free but to achieve a better everyday life”.

Traditionally, Norwegian secondary mental health services have concentrated mainly on symptom management. However, the IMR program has redirected this focus, enabling a collaborative exploration with patients on how to live optimally with symptoms, diagnoses, and illnesses, and lead dignified, meaningful lives. The following quote illustrates the practitioners’ awareness of that shift: “Yes, [IMR] makes it about life, not just getting rid of voices or symptoms. However, that’s not always easy when working in secondary mental health care services [that are traditionally illness-focused]” (P29). Building on this shift, practitioners cultivated an environment that enabled patients to become more self-reliant, namely by providing access to information, experience, and training in self-management skills. These empowered patients come to grips with their own illness and realize their own capacity for control. In this way, practitioners experienced that the IMR program supported patients in developing a set of versatile tools, a kind of toolbox. “In IMR, we give [patients] the means to deal with their suffering themselves” (P27). Practitioners reported experiencing an increased focus on delivering EBP that adhere to prescribed manuals. Some practitioners actively debated whether manual-based programs align with recovery-oriented practices, acknowledging the potential discrepancies between structured programs and the personalized recovery focus. Nevertheless, they were confident in the IMR program’s compatibility with recovery-oriented practices. One practitioner stated:

*“I think that IMR is person-centered. It’s about recovery. The focus is on the individual*,* and I used to say*,* “It’s you that controls the process*,* it’s you who makes the choices*,* and it’s only you who knows what’s important.” The whole program supports that orientation (.) So*,* I think that the program is extremely recovery oriented”. - P15*.

Practitioners highlighted the IMR program’s adaptability to individual needs, despite its structured 11-module curriculum. They specifically appreciated the individualized approach of the first module, “Recovery Strategies,” which ensures that the treatment aligns with each person’s unique needs, goals, and preferred approach from the outset. As a result, they found that the IMR program, despite being standardized, was tailored to meet each patient’s personal needs and objectives. They underscored that the key to practicing a manual-based program hinged on their application of the material and their attentiveness and responsiveness to the patient.

*“It depends on how you use the material*,* the framework*,* or the tool. I think that it doesn’t matter how recovery-oriented [the program] is if you as a person don’t listen or “tune in” to the person in front of you. So*,* I think that the most important thing is to “tune in” and be humble and responsive to what the patient conveys to you”. -P1*.

Although most practitioners emphasized that the recovery orientation of IMR is largely dependent on practitioners’ ability to personalize and adapt it to the unique needs and goals of each patient, some experienced a tension between the structured nature of IMR and the inherently personal and sometimes spontaneous process of recovery.

*“It’s an interesting*,* relevant critique*,* because it sheds light on paradoxes within the concept of recovery. Recovery is a unique personal process*,* right? But narratives from patients in recovery are rarely related to structured treatment. Sometimes things happen spontaneously*,* and a challenge that we’ve discussed is that when we try to systematize things that should be spontaneous*,* what happens then? Do we lose spontaneity?” - P15*.

In summary, IMR shapes practitioners’ approaches, enhancing their understanding and ability to help individuals live well despite challenges. They recognized the tension between the structured nature of the IMR program and the inherently personal and spontaneous process of recovery. This tension underscores the challenge of systematizing aspects of recovery that are often deeply personal. Practitioners strove to maintain the program’s integrity while making necessary adjustments to ensure it remained person-centered.

### IMR emphasizes a recovery-oriented approach in the delivery of services

This theme reflects the growing appreciation for recovery-oriented approaches in mental health service delivery. Practitioners stressed the necessity of enhancing the focus on recovery-oriented treatments within mental health services, driven by demands from health authorities and user organizations. While national public policies and guidelines may not explicitly reference IMR, practitioners perceive IMR as a potential solution to align with the shift towards recovery-orientation. As P29 put it; “In relation to the guidelines that highlight more recovery-oriented thinking in services, IMR covers that in a simple way. Offering the IMR program will help you [services] to stay recovery-oriented”. Nonetheless, practitioners delivering IMR faced challenges, especially in striking a balance between focusing on illness and wellness, as well as integrating individual, social, and health promoting perspectives within the program.

Practitioners emphasized that IMR addresses both the management of illness and the promotion of personal well-being. However, they acknowledged challenges in balancing these aspects. They noted that IMR placed a greater emphasis on wellness compared to other treatments, with a reduced focus on illness. Less attention to illness, diagnosis, and deficiency appealed to them because they thought these topics had received too much attention in secondary mental health services. If they were to work with patients to explore how they could live a dignified and meaningful life, despite their conditions, there was a need for a stronger focus on the patient’s strengths, resources, and equipping them with skills and tools that could enable them to be in the driver’s seat of their own lives. The shift from illness-focused to mastery-focused treatment underscores the importance of using conscious language that supports the treatment’s wellness orientation. Many had been delivering IMR for years—some for nearly a decade. Initially, there was no translated manual available, so they undertook translation work themselves. During this process, they aimed to incorporate what they perceived as strengths-based language, which they believed could align with recovery-oriented practices.

*“When we started IMR*,* the manual was only in American English. We had to find Norwegian words*,* preferably positively charged ones. In the American version*,* the program is called “Illness Management and Recovery*,*” but since we wanted to start a recovery group*,* we chose to translate it to “Individuell mestring og tilfriskning” [“Individual Coping and Recovery” in English]. - P23*.

Despite this increased focus on strengths-based language, the practitioners stated that the IMR manual still contained language and concepts that were not strengths-based to the extent they considered optimal. They concurred that the IMR program ought to provide factual information about illness, medication and treatment. However, they commented that some of the language used and the explanations in the program were more aligned with a medical paradigm than a recovery-oriented approach. As P1 pointed out:

*“We think that its conceptualizations of illness are imbued with medical thinking. But we still have limited knowledge of what schizophrenia is*,* how it occurs*,* and why some people experience it (.) However*,* even though [IMR] was founded on medical thinking*,* we can choose to tone that down which we have done”.*

Some practitioners advocated for IMR to be a dynamic program that adapted to changes in the field of mental health and society. Consequently, they wanted to adopt a Dutch version of the American manual. They believed this version aligned more closely with the Scandinavian environment and conditions, emphasized medical aspects less, and used strengths-based language more extensively. They were hopeful that this would help them strike a better balance between illness management and promoting wellness.

“P8: We have to make some adjustments in the IMR manual because the world keeps turning, somehow. The material is becoming outdated as new perspectives emerge.

P4: It [the Norwegian manual] needs to be revised, doesn’t it?

*P8: Yes*,* it’s on the table. But maybe we’ll choose to translate the Dutch version instead*,* because it aligns more with our mindset and is less American (.) The last time we went to a conference*,* we met someone from the Netherlands who was working on the Dutch Manual. It [Dutch Manual] has newer illustrations*,* less text and different language*,* which is perhaps the most important. Much more recovery-oriented language”.*

Practitioners advocated for the inclusion of more social and health-promoting perspectives into the IMR program. Some expressed concerns about the program’s reliance on models like the stress-vulnerability model, which predominantly focuses on individual pathology while dismissing the role of social factors. Instead, they proposed incorporating what they termed “social models”, which emphasize the importance of social conditions for an individual’s mental health. One practitioner (P2) highlighted: “Yes, the stress and vulnerability model is a model among many others. Unfortunately, social models are given less space, as are social explanations and causes of mental health problems”. Practitioners underscored the importance of wellness within the program, emphasizing elements that fosters good health, enhance well-being, and instill hope. They recognized social forces (e.g., living conditions, work, finances) – significantly contribute to the onset and persistence of mental illness.

“P1: I want hope to be more integrated [in the program]. We’re supposed to be emphasizing that, but I think that attention to hope could be sharpened.

P2: The same goes for the salutogenic view.

*P1: Yes*,* and to be more aware of seeing [patients’] potential and resources and to work even more toward that”.*

Furthermore, practitioners suggested more attention to social determinants of health (SDH) that greatly affect health and well-being. They called for decisive action to address living conditions, finances, work, housing, and inclusion to support dignified, meaningful community living for those with mental illness. While some modules briefly address these aspects, practitioners believe they lack sufficient coverage throughout the program. They suggest that SDH deserves as much exploration as illness, advocating for a more equitable balance between addressing individual explanations and social and societal factors. P2 emphasized this need for greater balance:

*“Yes*,* we already know what kinds of things put people in better positions to take care of themselves: networks*,* family relationships*,* finances*,* not necessarily being rich but having enough to live comfortably*,* having a safe place to live*,* etc. But place of residence is hardly mentioned at all [in the manual]*,* even though it’s basic for any recovery process”.*

In summary, the practitioners recommended a more robust integration of social aspects into the IMR program, extending beyond mere individual aspects. They argued for more of a strengths-based approach that would balance illness and wellness information, minimize focus on the medical model, highlight hope and well-being, and address social determinants of health.

## Discussion

Our results underscored that IMR is a dynamic program that helps mental health practitioners support patients in learning how to manage their conditions and lead more meaningful lives. Despite its structure and extensive curriculum, practitioners can tailor IMR to their own needs by aligning recovery-oriented principles. However, the practitioners desire more social and health-promoting perspectives in the program, including a greater emphasis on strengths-based language, the promotion of wellness, and the awareness of the social determinants of health. With IMR as a backdrop, we further explored the relationship between EBPs and ROPs, the balance between illness management and wellness promotion, and viewing recovery as both an individual and a social process.

Practitioners appeared to see no contradiction between providing IMR as EBP and as an ROP in secondary mental health services. This aligns with the view of Farkas et al. [14] that EBPs can be compatible with recovery if the program dimensions are consistent with the four key recovery values: person orientation, person involvement, self-determination, and personal growth. In fact, practitioners in this study found that IMR effectively identified and incorporating the goals, interests, and strengths of a person into their treatment, maintaining a focus on recovery goals while fostering patient autonomy. Contrary to Karlsson and Borg’s [21] argument that EBPs are rigid and lack the personalization required for ROPs, practitioners have stressed the importance of customizing treatment to the individual patient in all interventions, including EBPs such as IMR. This customization was seen as being linked to the practitioner’s behavior and their ability to deliver services in line with recovery values. Mueser [16] has argued that EBPs should be seen as a set of tools or a ‘human technology’ for achieving important treatment goals, rather than having any inherent value. They are neither inherently recovery-oriented nor opposed to recovery. However, when the delivery of these practices is customized to the individual and guided by key recovery values, they can be a powerful tool for helping patients pursue their personal goals.

Similar to the findings of Davidson et al. [13], we have found that practitioners experienced IMR and ROPs as complementary rather than conflicting. While the analogy of oil and vinegar suggests they are separate entities that can be combined fruitfully, it is important to note that IMR can be implemented as an ROP. Practitioners did not view them as fundamentally different types of services that could not be blended or integrated, but as components that, when combined, could create a “tasty vinaigrette.” This blend was achieved by drawing out and enhancing the best aspects of each approach, creating a whole that is greater than the sum of its parts. EBPs are based on the ethical obligation to offer effective treatments to people with mental illness. In the absence of empirical evidence, practitioners are unable to guarantee the provision of optimal care, thereby impeding patients‘ ability to make decisions that are well-informed [13]. The introduction of recovery as a concept in the mental health field has shifted the focus from on symptom reduction to personal growth and living a meaningful life with mental illness [62, 63]. Davidson et al. [13] highlighted the novelty of the recovery concept. As ROPs develop, it is crucial to integrate EBPs to ensure these new approaches are effective and avoid potential missteps. Integrating the principles of EBPs with those of ROPs might necessitate expanding the definition of evidence to encompass first-hand experience and upholding the patient’s active role in treatment. Together, these approaches can efficiently guide patient care [64], incorporating recovery values [14] and people’s lived experiences. Practitioners in our study found that delivering the evidence-based IMR program helped integrate ROP into the system of specialized mental health care. Linking EBP to mental health recovery clarified what ROPs entail in daily clinical practice.

Practitioners reported experiencing an imbalance between managing illness and fostering wellness in the IMR program. Despite recognizing the program’s recovery orientation, some practitioners perceived that the medical underpinning in IMR was too strong. This perception was influenced by their understanding of mental illness, the treatment forms mentioned, and the language used in the manual. While acknowledging the importance of addressing biological/medical factors for providing valuable information pertinent to illness management, they felt that these factors should not overshadow social and psychological factors. This could either reflect the manual itself or result from the practitioners’ interpretation of it. Beresford et al. [65] emphasized the continued dominance of models favoring biological factors over social and psychological ones in services, despite the growing interest in recovery. Practitioners moderated elements they perceived as conflicting with a recovery-oriented approach, such as softening language that did not emphasize strengths and avoiding frameworks that were overly biological or narrow-minded. This adjustment aimed to balance the valuable insights from the stress–vulnerability model with a more holistic understanding of mental health, incorporating additional social and psychological factors. By doing so, they aligned the program more closely with their recovery-oriented objectives, ensuring a comprehensive approach to patient care.

Practitioners acknowledged the critical role of illness management when working with people struggling with serious mental illness yet critiqued the program’s limited and individual-centric explanations. They called for more comprehensive explanations that incorporate the social aspects of living with mental illness. In line with Topor et al. [6, 10] and Alegría et al. [66], practitioners emphasized the importance of understanding the impact of social factors on mental health and advocated for a balanced focus on both personal and societal influences on vulnerability. The limitations of the stress–vulnerability model, which some practitioners found to be overly simplistic, became apparent. This model strongly emphasizes individual factors and their interaction with stress in influencing the onset and course of mental illness. Although it acknowledges environmental and social factors, practitioners did not view these as the primary focus, potentially due to their perceived complexity and being outside the individual’s control. This perspective aligns with Demke’s [67], criticism, where the model is faulted for neglecting social factors and adhering to a medicalized view that isolates the individual, despite claims of integrating social dimensions. Echoing Goldblatt et al. [68], practitioners stressed the importance of understanding social inequality and its impact on people’s lives. Practitioners advocated for integrating insight into the significant influence of social factors on health, happiness, and recovery, proposing that these insights be integrated into the program and for actions to address these disparities. Given the multifaceted nature of social factors, it might be more feasible and less controversial to incorporate them into the stress–vulnerability model rather than overhauling the entire IMR program. This approach could face less resistance and be easier to implement. Alternatively, integrating the stress-vulnerability model with the social determinants of health could provide a more comprehensive understanding of mental illness in the IMR program, helping to identify social factors that contribute to individual vulnerabilities and vice versa.

Practitioners expressed concern about the perceived imbalance in the IMR program, where a strong emphasis on personal recovery tends to overshadow the individual’s social context. However, recovery and optimal functioning require attention to larger social factors and forces. According to Topor et al. [6], viewing recovery solely as an individual journey, rather than recognizing its social dimensions – may lead to acceptance of the limitations inherent in an illness-based model. This study suggests that greater attention should be given to individuals within their context, examining social structures and conditions that may hinder or facilitate the recovery process. Similarly, Huggard et al. [69] emphasize the importance of recognizing the consequences of overemphasizing biological determinants, which can affect client-practitioner relationships and perpetuate stigmatizing attitudes. It is necessary to examine whether this view is common among mental health practitioners in other countries delivering IMR treatment. Scandinavian researchers often advocate for recovery as both an individual and social process, emphasizing the importance of social factors in understanding the onset and maintenance of mental illness. This focus reflects the strong tradition in Scandinavian countries of emphasizing social determinants of health, differing from other countries with different welfare models. In Norway, social inequality in mental health is a growing concern. Comprehensive strategies and active social policies proposed to address this disparity in mental health services are viewed as important tools for reducing health inequalities and promoting recovery [70].

In Norwegian secondary mental health services have traditionally leaned toward illness-oriented approaches to care [11]. This focus can limit the acceptance of treatments that deviate from bio-medical principles. Medical treatments that emphasize medical history, pathology, diagnosis, and symptom reduction differ significantly from approaches that focus on promoting health and wellness, exploring patients’ own resources, choices, and coping strategies, as well as examining how social structures and determinants contribute to the onset and maintenance of individual challenges [71]. Despite this divergence, the IMR program seeks to bridge traditional views of illness with the concept of mental health recovery. It does this through a dual aim: to improve coping skills and clinical outcomes for symptom management, and to enable individuals to lead fulfilling lives by setting and achieving personal recovery goals [25, 26]. Given that IMR has this bipartite goal, linked to clinical and personal recovery, there will naturally be elements of biological and individual understandings, explanations and language in the program. The program’s foundation on comprehensive research [26] has likely provided necessary acceptance within Norwegian secondary mental health services. Its strong emphasis on empowering individuals to manage and take control of their lives implies that mental health practitioners should act more as consultants rather than authoritative experts, this latter being a role too often seen in secondary mental health services. Including more social factors in the program will require a willingness to incorporate these factors, in addition to strengthening the evidence related to social factors in the lives of people struggling with mental illness.

### Strengths and limitations

This study’s strengths feature its inclusion of mental health practitioners with diverse educational backgrounds and work experience, recruited from five different mental hospitals and district psychiatric centers across Norway. This diversity enhances the potential for broad and varied perspectives on the IMR program.

However, our study sample was imbalanced in terms of gender and profession, with an overrepresentation of women and mental health nurses, reflecting the general composition of mental health workers in Norway. Additionally, although discussions with co-authors were integral throughout the analysis process, the initial analysis of data was performed solely by the first author.

The five authors of the study come from diverse professional backgrounds, including psychologists, family therapists, a social worker, and a teacher, all with extensive experience in mental health services and research. This diversity provided multiple perspectives on the data, enhancing the analysis and ensuring reflexivity. These discussions validated ideas, explored various assumptions, and facilitated data interpretation. Furthermore, this collaborative approach extended to crafting a coherent narrative and producing the manuscript.

## Concluding remarks and clinical implications

Practitioners were generally satisfied with IMR as a psychosocial EBP and that delivering IMR provided them with an opportunity to implement ROPs in the context of secondary mental health services. ROPs and EBPs are often seen as mutually exclusive, similar to medical and psychiatric versus psychosocial approaches in mental health care. Despite their different foundations, we suggest that future discussions in clinical practice and research should focus more on how those practices and programs can be complementary. We argue that IMR has the potential to build bridges between traditional psychiatric rehabilitation and mental health recovery and between ROPs and EBPs. As a manual-based program, IMR provides an opportunity to implement ROPs in the context of secondary mental health services. Mental health practitioners are provided with a tangible, structured program in which they can work systematically with topics in the manual while centering the individuals and tailoring the sessions to their aspirations, goals, and choices. Based on our results, we argue that ROPs and EBPs are complementary rather than conflicting, which increases the likelihood of including recovery-oriented outcome measures in EBPs. Expanding evidence-based outcome measures to include recovery-oriented outcomes, should be welcomed, not dismissed, as long as the individual’s experiences and role as a partner are recognized.

Based on our findings, there is a need to balance individualistic and social perspectives in IMR. Increasing contextual understandings and the roles of social aspects to promote recovery aligns with previous research and theory that critiques individualistic approaches to recovery [6, 7, 72, 73]. Rather than discontinuing IMR due to its medical or individualistic recovery emphasis, practitioners should strive to enhance its wellness-orientation, contextual understandings, social aspects, and strengths-based language. Furthermore, engaging in dialogue with the manual’s founders to suggest adaptations and updates could promote a more balanced approach to wellness and social determinations in future updated versions of the IMR manual.

We advocate for the consideration of practitioners’ critique that the individualistic recovery perspective in IMR overshadows social aspects. Therefore, future iterations of IMR and other evidence-based recovery-oriented programs should neither underestimate nor overlook the importance of social recovery.

## Supplementary Information

Supplementary Material 1.

## Data Availability

Excerpts from the interview transcripts relevant to the study have been included in the paper. Unfortunately, a complete, anonymized data set cannot be shared due to individual privacy concerns and the lack of consent from participants. However, we can provide portions of the transcripts that are relevant to this paper. If other researchers wish to access the data, obtaining new consent from the participants will be necessary. For additional information regarding the data, please contact the corresponding author at kristin.b.orjasater@nord.no.

## Abbreviations

EBP: Evidence-Based Practice
IMR: Illness Management and Recovery
ROP: Recovery-Oriented Practice
