# Supplementary material for: The illness management and recovery program: a contribution to recovery-oriented secondary mental health services
Source: BMC Health Serv Res. 2025 May 24;25:750. doi: 10.1186/s12913-025-12907-2 (PMC12102869; doi:10.1186/s12913-025-12907-2)
Supplement: Supplementary file 1 — Supplementary Material 1. [file 12913_2025_12907_MOESM1_ESM.docx]

**Discussion Guide**

The interview guide serves both as preparation and a roadmap for conducting focus group interviews. It encompasses themes and questions that the researcher will investigate to address the research question. The aim is to conduct focus group interviews that are as flexible and conversational as possible, rather than rigidly structured. Before the interview, researcher will:

- Briefly introduce the research project
- Explain how the data will be used and presented
- Offer a concise overview of the focus group as a method of interviewing
- Clarify the participants’ research ethical rights and the researcher’s commitment to confidentiality and anonymization of the data.

**Questions**

1. Please share your professional background and work experience.
2. From your perspective, could you describe what IMR is and the aim of the treatment program?
3. What do you perceive as the core of IMR?
4. What was the background for you starting with IMR?
5. Can you share how you work with IMR at your facility?
6. What framework and/or conditions are necessary to run IMR?
7. What is required of you as staff to run IMR?
8. How do you, as staff, experience working with IMR?
9. Could you share experience or thoughts on how IMR treatment aligns or conflicts with your understanding of treatment, roles, values, and flexibility?
10. What do you experience as the strengths of IMR?
11. What do you experience are the weaknesses of IMR?
12. Before we conclude, is there anything related to IMR that you want to elaborate on?
